# Shear Banding in 4:1 Planar Contraction

**DOI:** 10.3390/polym11030417

**Published:** 2019-03-04

**Authors:** Soroush Hooshyar, Natalie Germann

**Affiliations:** Fluid Dynamics of Complex Biosystems, School of Life Sciences Weihenstephan, Technical University of Munich, 85354 Freising, Germany; soroush.hooshyar@tum.de

**Keywords:** contraction flow, polymer solutions, shear banding, two-fluid model, nonequilibrium thermodynamics

## Abstract

We study shear banding in a planar 4:1 contraction flow using our recently developed two-fluid model for semidilute entangled polymer solutions derived from the generalized bracket approach of nonequilibrium thermodynamics. In our model, the differential velocity between the constituents of the solution allows for coupling between the viscoelastic stress and the polymer concentration. Stress-induced migration is assumed to be the triggering mechanism of shear banding. To solve the benchmark problem, we used the OpenFOAM software package with the viscoelastic solver RheoTool v.2.0. The convection terms are discretized using the high-resolution scheme CUBISTA, and the governing equations are solved using the SIMPLEC algorithm. To enter into the shear banding regime, the uniform velocity at the inlet was gradually increased. The velocity increases after the contraction due to the mass conservation; therefore, shear banding is first observed at the downstream. While the velocity profile in the upstream channel is still parabolic, the corresponding profile changes to plug-like after the contraction. In agreement with experimental data, we found that shear banding competes with flow recirculation. Finally, the profile of the polymer concentration shows a peak in the shear banding regime, which is closer to the center of the channel for larger inlet velocities. Nevertheless, the increase in the polymer concentration in the region of flow recirculation was significantly larger for the inlet velocities studied in this work. With our two-fluid finite-volume solver, localized shear bands in industrial applications can be simulated.

## 1. Introduction

Contraction flow is of great importance in many processing operations, such as molding and extrusion of viscoelastic materials. Furthermore, the 4:1 planar contraction is a suitable benchmark problem for the evaluation of new models or codes. Different types of vortices, namely, salient and lip vortices, can appear in this geometry. The lip vortex originates from the re-entrant corner and dominates the flow by vortex enhancement and growth [1]. Experimental evidence has revealed that the vortex enhancement is absent for Boger fluids while it is apparent for shear-thinning fluids [2,3]. Comparisons between strain-hardening low-density polyethylene (LDPE) and strain-softening polystyrene suggest that the size and strength of the vortices are influenced by both extensional and shear properties [4,5]. With increasing flow rate, the vortex size increases if the ratio of the extensional to the shear viscosity increases and vice versa [6].

Numerical simulations of viscoelastic contraction flow use constitutive models according to the material under investigation. Olsson [7] observed the lip vortex of a shear-thinning fluid for the first time using the Giesekus model. In several works, the Oldroyd-B and Phan-Thien/Tanner (PTT) models were utilized to describe the behavior of Boger and shear-thinning fluids, respectively. The Oldroyd-B model predicts that the size of the salient vortex decreases, and the lip vortex appears and further grows with increasing flow rate [3,8]. The results of Aboubacar et al. [6,9] revealed that by increasing the elasticity, the Oldroyd-B model and both the linear and exponential versions of the PTT model with a small value of the parameter simultaneously controlling shear-thinning and strain hardening exhibit vortex reduction. The PTT model reverts to the Oldroyd-B model if the value of this parameter reduces to zero. However, the size of the vortex increases for the PTT model with stronger shear-thinning behavior. Thompson et al. [10] proposed a new constitutive equation that can predict the increase in the corner vortex size with strain hardening. The idea of their model was that the stress tensor is an isotropic function of the strain rate and the relative rotation rate. White and Baird [11] used the PTT model and suggested that extension hardening increases the size and intensity of the vortex, and this proposition was in agreement with their flow visualization and birefringence data [12]. The Pom-pom model is an appropriate model to describe contraction flows due to shear-thinning and bounded strain hardening-softening properties. Many works have adopted this model and showed that the size of the vortex increases with the ratio of the extensional to shear stresses [13,14,15]. Ferrás et al. [16] used the PTT model along with slip boundary conditions. Increasing the slip enlarges the lip vortex until it absorbs the salient vortex. The new single vortex grows in size and intensity with the value of the slip coefficient.

Many features of a planar contraction flow cannot be observed in the 4:1 geometry. Alves et al. [17] used the linear PTT model to numerically study the effects of the contraction ratio (CR) and the Deborah number (DE). They illustrated in a map that the lip vortex appears at De≈1−2, the vortex enhancement starts at De/CR≈0.5−1, and the lip vortex becomes completely dominant for De/CR≥1−2. Their results are in qualitative agreement with the visualizations of Evans and Walters [18]. The material properties of a fluid also affect the vortex enhancement; for instance, no lip vortex was experimentally found at CR=4 for Boger fluids (1.0 wt/wt% polyacrylamide dissolved in maltose syrup and water) or for 0.3 and 0.5 wt/wt% shear-thinning aqueous polyacrylamide solutions observed by Evans and Walters [19]. However, a lip vortex was observed for the lower concentration of 0.2 wt/wt%. This result was numerically confirmed using the finite extensible nonlinear elastic with Peterlin’s closure (FENE-P) model [20].

Shear banding is a ubiquitous phenomenon observed in soft materials, such as semidilute entangled polymer solutions, and is defined as the formation of localized bands with different shear rates. However, limited information is available about its origin and the impact on processing. Hemminger et al. [21] experimentally studied a 4:1 rounded-corner contraction flow of 75 kbp DNA solutions with concentrations from 0.1 to 1.0 wt/wt% over a wide range of Deborah numbers (up to $2\times 10^{4}$ for the most concentrated solution). They observed that the vortex flow dominates for the non-shear-banding solutions with concentrations of 0.1 and 0.5 wt/wt%. However, shear banding was found for the higher concentrations of 0.7 and 1.0 wt/wt% at the entrance of the contraction with a high flow rate at the centerline and a low flow rate at the corner. The authors found that in this regime, reduction in slip length, obtained by, for example, increasing the solvent viscosity, causes the vortex flow to become dominant again. As the phenomenon of shear banding was studied in the converging zone before the contraction, the flow velocities were much higher than those examined in the present article.

A kinetic theory model [22,23] and a nonequilibrium thermodynamic model [24] were recently proposed for shear banding polymer solutions. These models are built on the hypothesis that shear band formation is associated with shear-induced migration. As opposed to the standard one-fluid polymer models, not only steady-state velocity banding but also banded concentration profiles can be predicted if a realistic constitutive equation for the conformation/stress tensor is used. In the two-fluid framework employed by Hooshyar and Germann [24], the differential velocity is treated as a state variable. This description is advantageous since it simplifies the specification of the boundary conditions. However, an explicit expression is better for convergence in some cases. The behavior of this model was analyzed in a cylindrical Couette flow [24] and a pressure-driven channel flow [25]. The results confirmed that the steady-state banded solution is caused by shear-induced migration, unique for different initial conditions, and independent of deformation history. The profile of the volumetric flow rate along the channel calculated for different values of the pressure gradient shows a spurt. Because the steady flow curve is monotonic under homogeneous conditions, no hysteresis was observed for the ramp-up and ramp-down tests. More details about the theoretical foundations of these two models are provided in our previous two papers Hooshyar and Germann [24,25]. Recent theoretical and experimental developments in the area of shear banding entangled polymer solutions were comprehensively reviewed in [26].

Hitherto, no two-fluid model for shear banding polymer solutions has been solved for 4:1 planar contraction flow. The goal of this work is to study the new model in this geometry and to investigate the influence of the shear bands on the vortices. The remainder of this paper is organized as follows. In Section 2, we provide the model equations. Section 3 introduces the flow problem and numerical procedure. In Section 4, we analyze the computational results. The conclusion is presented in Section 5.

## 2. Polymer Model

In this section, we present the two-fluid model developed by Hooshyar and Germann [24] for semidilute entangled polymer solutions. For the polymer species, the state variables are the mass density $\rho_{p}$; the momentum density $m^{p}=\rho_{p}v^{p}$, with $v^{p}$ being the velocity; the number density $n_{p}=\left(\rho_{p}/M_{p}\right)N_{A}$, where $M_{p}$ is the molecular weight of the polymer and $N_{A}$ is the Avogadro constant; and the conformation density tensor $C=n_{p}c$, with c representing the average second moment of the end-to-end connection vector of the polymeric constituents. The state variables of the model for the solvent are defined as the mass density $\rho_{s}$ and the momentum density $m^{s}=\rho_{s}v^{s}$, with $v^{s}$ being the velocity. The time evolution equations of the state variables are as follows:

(1) $$\begin{array}{cc} \rho \frac{\partial v}{\partial t} & =-\rho v\;\cdot \;\nabla v-\nabla p+\nabla \;\cdot \;\sigma , \end{array}$$

(2) $$\begin{array}{cc} \frac{\rho_{p}\rho_{s}}{\rho } & \left(\frac{\partial }{\partial t}+v\;\cdot \;\nabla \right)\left(\Delta v\right)=\frac{\rho_{s}}{\rho }\left\{-\nabla \left(n_{p}k_{B}T\right)+\nabla \;\cdot \;\sigma^{p}\right\}\\ & -\frac{\rho_{p}}{\rho }\left\{-\nabla \left(n_{s}k_{B}T\right)+\eta_{s}\nabla^{2}v^{s}\right\}-\frac{G_{0}}{D}\Delta v\;, \end{array}$$

(3) $$\begin{array}{cc} \frac{\partial n_{p}}{\partial t} & =-\nabla \;\cdot \;\left(v^{p}\;n_{p}\right), \end{array}$$

(4) $$\begin{array}{cc} \frac{\partial C}{\partial t} & =-\nabla \;\cdot \;\left(v^{p}C\right)+C\;\cdot \;\nabla v^{p}+{\left(\nabla v^{p}\right)}^{T}\;\cdot \;C\\ & -\frac{1}{\lambda_{1}}\left[\left(1-\alpha \right)I+\alpha \frac{K}{n_{p}k_{B}T}C\right]\;\cdot \;\left(C-\frac{n_{p}k_{B}T}{K}I\right)\\ & +\frac{1}{\lambda_{2}}{\left[\mathrm{tr}(\frac{K}{n_{p}k_{B}T}C)-3\right]}^{q}\left(C-\frac{n_{p}k_{B}T}{K}I\right)\\ & +D_{nonloc}\left(C\;\cdot \;\nabla \left(\nabla \;\cdot \;\sigma^{p}\right)+{\left[\nabla \left(\nabla \;\cdot \;\sigma^{p}\right)\right]}^{T}\;\cdot \;C\right). \end{array}$$

Equation (1) is the Cauchy momentum balance, where v is the total average velocity of the solution, *t* is the time, $\rho =\rho_{p}+\rho_{s}$ is the total mass density, *p* is the pressure, and σ is the extra stress. Equation (2) is the time evolution equation of the differential velocity Δv. Here, $k_{B}$ is the Boltzmann constant, *T* is the absolute temperature, $\eta_{s}$ is the viscosity of the solvent, and $\sigma^{p}$ is the extra stress associated with the polymer. The divergence of $\sigma^{p}$ accounts for the stress-induced migration. The spatial gradients of the variables $n_{p}$ and $n_{s}$ describe the Fickian diffusion. The local diffusivity constant *D* controls the diffusion between the polymeric constituents and the solvent. The value of *D* does not affect the steady-state solution. As the variation in the polymer concentration is very small here, Equation (2) is difficult to solve with a lower-order discretization method. Therefore, we neglected the left-hand side and used the value of the previous iteration for the calculation of the Laplacian term. Equation (3) is the time evolution equation of the polymer number density. The terms of this equation constitute a material derivative, which accounts for the fact that the profile of the polymer concentration can be inhomogeneous. Equation (4) is the time evolution equation for the conformation density tensor C. The left-hand side and the first three terms on the right-hand side form the upper-convected time derivative. To have better convergence, we reformulated the model equations in terms of the conformation density tensor. This results in a convective term of divergence form, which can be discretized using finite volumes in a straightforward manner. The fourth term on the right-hand side of Equation (4) is the Giesekus relaxation, with α being the anisotropy factor. The fifth term on the right-hand side is an additional nonlinear relaxation term. This term is similar to the one used in the Rolie-Poly model accounting for convective constraint release (CCR) and chain stretch [27]. The power-law pre-factor ${\left[K/\left(n_{p}k_{B}T\right)\mathrm{tr}C-3\right]}^{q}$, where *K* is the Hookean spring constant of the polymer, captures the upturn of the flow curve at high shear rates. This term is a scalar function of the trace of the conformation tensor and thus a measure for polymer stretch [24]. The last term on the right-hand side of Equation (4) controls the smoothness of the profiles and guarantees a unique solution, where $D_{nonloc}$ is the nonlocal diffusivity constant.

The preceding set of time evolution equations is closed by an explicit expression for the extra stress:

(5) $$\begin{array}{cc} \sigma = & \sigma^{p}+\sigma^{s}=KC-n_{p}k_{B}TI+\eta_{s}\left[\nabla v^{s}+{\left(\nabla v^{s}\right)}^{T}\right], \end{array}$$

where the first term on the right-hand side accounts for the viscoelastic contribution of the polymer, and the second term accounts for the viscous stress of the solvent. To calculate the phase velocities of the polymer and the solvent, the total average and differential velocities can be used as follows:

(6) $$\begin{array}{cc} v^{p}= & v+\frac{\rho_{s}}{\rho }\Delta v, \end{array}$$

(7) $$\begin{array}{cc} v^{s}= & v-\frac{\rho_{p}}{\rho }\Delta v. \end{array}$$

In the equilibrium state of rest, where v=0 and Δv=0, the analytical solution of $n_{p}=n_{p}^{0}$ and $C=\left(n_{p}^{0}k_{B}T/K\right)I$ can be obtained.

## 3. Numerical Method

**Figure 1:**
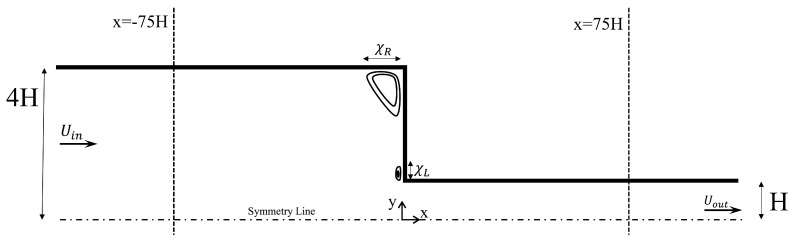
Planar 4:1 contraction geometry.

We solved the model equations (Equations (1)–(5)) for a steady, laminar, incompressible, two-dimensional flow through a 4:1 planar contraction. A schematic sketch of the flow geometry is given in Figure 1. The half-width of the downstream channel is denoted by the characteristic height *H*. As required by the problem, the half-width of the upstream channel is 4H. Inlet and outlet effects can be neglected since we assume 100H for both the lengths of the upstream and downstream channels. The Deborah number is defined as $De=\lambda_{1}U_{out}/H$, where $U_{out}$ is the mean velocity at the outlet. The Reynolds number is defined as $Re=\rho U_{out}H/\eta_{0}=E^{-1}/De$, with *E* and $\eta_{0}$ being the elasticity number and the zero shear viscosity, respectively, defined below. The Cartesian coordinate system was used as the reference frame. Any dependence on the z-direction was ignored for simplicity.

In the following, we work with non-dimensional quantities. The location is scaled by the characteristic height, $\tilde{y}=y/H$; the time is scaled by the characteristic relaxation time, $\tilde{t}=t/\lambda_{1}$; the extra stress is scaled as $\tilde{\sigma }=\sigma /G_{0}$; and the conformation tensor associated with the polymer is scaled as $\tilde{C}=\left(K/n_{p}k_{B}T\right)C$. The number densities of the polymer and the solvent are normalized using the values at equilibrium as ${\tilde{n}}_{p}=n_{p}/n_{p}^{0}$ and ${\tilde{n}}_{s}=n_{s}/n_{p}^{0}$, respectively. The dimensionless parameters with respect to these scalings are the elasticity number $E=G_{0}\lambda_{1}^{2}/\rho H^{2}$; the ratio of the molecular weight of the solvent to that of the polymer, $\chi =M_{s}/M_{p}$; the viscosity ratio $\beta =\eta_{s}/\eta_{0}$, with $\eta_{0}=G_{0}\lambda_{1}$ being the zero shear viscosity; and the ratio of the characteristic relaxation times $\epsilon =\lambda_{1}/\lambda_{2}$. The total polymer concentration corresponds to the initial uniform polymer concentration and is given in weight percent by $\mu ={\tilde{n}}_{p}^{0}/\left({\tilde{n}}_{p}^{0}+\chi {\tilde{n}}_{s}^{0}\right)$. The dimensionless diffusion coefficients are $\tilde{D}=D\lambda_{1}/H^{2}$ and ${\tilde{D}}_{nonloc}=D_{nonloc}\lambda_{1}/H^{2}$. The non-dimensional form of the model equations can be found in Appendix A.

The OpenFOAM v4.0 finite volume package, together with the viscoelastic solver rheoTool v.2.0 [28], was used to solve the flow problem. To add our model to the solver, we implemented the two-fluid description by using the differential velocity as an intermediate variable, similar to the approach of Guo et al. [29]. In our simulations, the convection term was discretized using the high-resolution Cubista scheme following a component-wise and deferred correction approach. The diffusion term and the gradients of the velocity and pressure were discretized using the Gauss linear scheme. The Crank–Nicolson method was employed for time discretization. The discretized flow problem was iteratively solved using the semi-implicit method for the pressure linked equations-consistent algorithm with 10 inner iterations per time step. The conjugate gradient method with a diagonal incomplete-Cholesky preconditioner was used for solving the continuity and momentum equations and the biconjugate gradient solver with an incomplete lower-upper decomposition for the remaining linear equations. The absolute tolerance for the variables was $1.0\times 10^{-7}$ for the steady-state test simulations.

At the inlet boundary, a uniform profile of the polymer number density and a uniform velocity field $U_{in}$ were imposed, resulting in a zero tensorial value for the stress and unity for the polymer conformation. At the outlet boundary, we assumed a vanishing pressure field and fully developed flow with Neumann conditions for the total velocity, the polymer number density and conformation, and the extra stress. At the solid walls, we utilized no-slip and no-flux conditions for the total velocity. For the polymer number density, we used the Neumann condition. The conformation and stress tensors were linearly extrapolated along the walls using the linearExtrapolation boundary available in rheoTool. The differential velocity must be zero for the conditions specified above. Since no asymmetry was observed relative to the channel centerline in our preliminary calculations, we solved the flow only for the upper half of the channel to avoid unnecessary computations, and we correspondingly used symmetry boundary conditions for the centerline.

The flow was solved using the four meshes reported in Table 1. The coarsest mesh M1 is obtained by considering the upper half of the mesh M1 utilized by Pimenta and Alves [30]. Their mesh is generated so that the resolution is higher near the walls and the corners. We doubled and tripled the number of faces of our mesh M1 in both spatial directions to obtain meshes M2 and M3, respectively. The number of faces of M3 is doubled in both directions to generate the mesh M4.

## 4. Results

**Figure 2:**
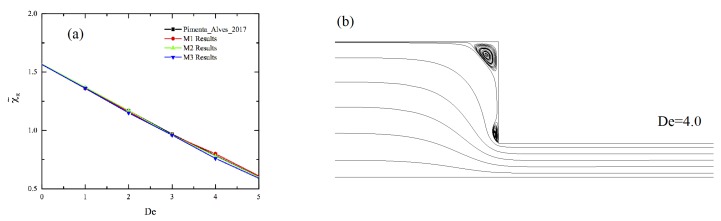
Solution of Oldroyd-B model for 4:1 contraction: (**a**) size of dimensionless corner vortex for different Deborah numbers and (**b**) streamlines at De=4 and Re=0.01. The results are compared with those of Pimenta and Alves [30] for validation.

To validate the numerical scheme, we first checked the consistency of the implemented terms of the new model by comparison with the numerical solution obtained for steady homogeneous Couette flow [24]. Afterward, we solved the benchmark problem for the Oldroyd-B model and compared our results with those of Pimenta and Alves [30]. The dimensionless size of the corner vortex, ${\tilde{\chi }}_{R}$, is shown in Figure 2a for Deborah numbers up to 4, where Re was kept constant at 0.01. As the Deborah number increases, the corner vortex becomes smaller. We find that the agreement is excellent. The streamlines displayed for De=4 in Figure 2b also closely match with those published for the same condition in Pimenta and Alves [30]. After the grid independence test, subsequent simulations were performed using mesh M3.

**Figure 3:**
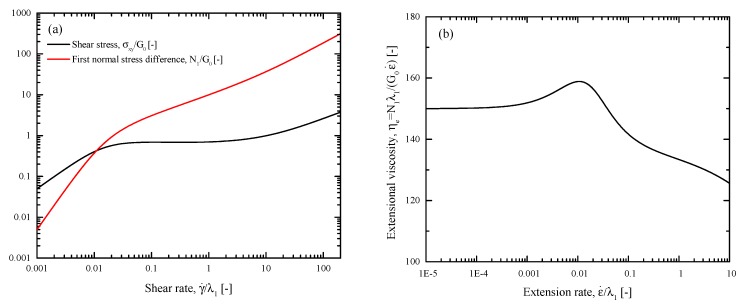
(**a**) Dimensionless shear stress and first normal stress difference vs dimensionless shear rate in homogeneous simple shear flow. (**b**) Dimensionless viscosity vs dimensionless extension rate in homogeneous uniaxial extension.

The planar contraction flow is a combination of a simple shear flow and a uniaxial extension. The predictions of our model for these flows under homogeneous flow conditions are shown in Figure 3. The values are the same as those used in our previous two papers [24,25]. The model parameters were determined by fitting the flow curve of the shear stress against the shear rate with shear experiments of a 10 wt/wt% 1.6 M shear-banding polybutadiene solution [31]. The parameters are the mobility factor α=0.73, the ratio of the characteristic relaxation times ϵ=0.0025, the power-law factor q=1.46, the viscosity ratio $\beta =10^{-4}$, and the ratio of the molecular weight of the solvent to that of the polymer $\chi =10^{-1}$. A moderate non-dimensional nonlocal diffusion coefficient ${\tilde{D}}_{nonloc}=10^{-3}$ was selected to remove the sharp kink in the banded profiles, and a moderate non-dimensional local diffusion constant $\tilde{D}=10^{-3}$ was used to avoid long running times since the value of this coefficient does not affect the steady-state solution. In homogeneous simple shear (Figure 3a), both the shear stress and the first normal stress difference increase monotonically with the shear rate. In homogeneous uniaxial extension (Figure 3b), the extensional viscosity shows a plateau followed by mild extension thickening and thinning.

**Figure 4:**
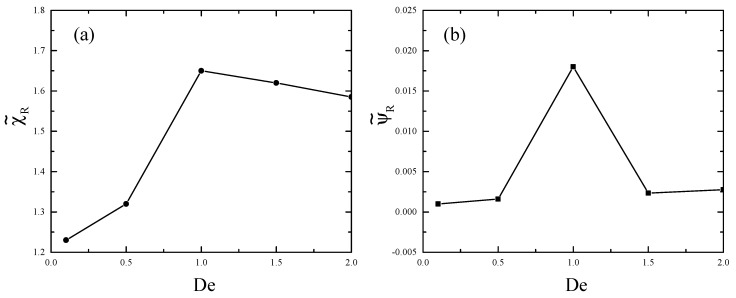
(**a**) Corner vortex size normalized by characteristic height *H* and (**b**) corner vortex intensity normalized by $U_{out}H$ versus Deborah number.

In Figure 4a,b, we show the size of the corner vortex, ${\tilde{\chi }}_{R}$, and the maximum value of the vortex intensity, ${\tilde{\psi }}_{R}$, respectively, for the Deborah numbers 0.1, 0.5, 1, 1.5, and 2. The corner vortex size increases with De due to shear-thinning as expected [6,32]; however, it decreases as the shear banding starts to occur at De=1, which is in agreement with the experimental data [21]. The vortex intensity follows the trend of the vortex size.

**Figure 5:**
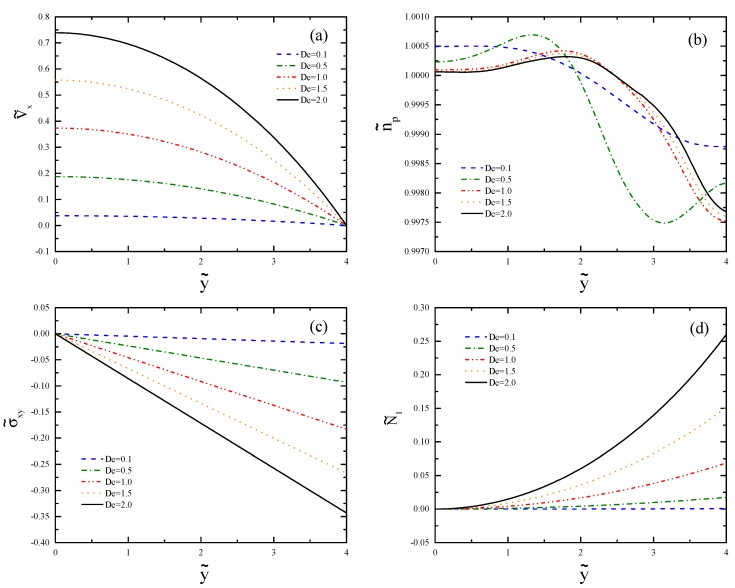
Profiles of (**a**) axial velocity, (**b**) polymer number density, (**c**) shear stress, and (**d**) first normal stress difference evaluated at $\tilde{x}=-75$ for different Deborah numbers.

In Figure 5, we see the profiles of the axial velocity ${\tilde{v}}_{x}$, the polymer number density ${\tilde{n}}_{p}$, the shear stress ${\tilde{\sigma }}_{xy}$, and the first normal stress difference ${\tilde{N}}_{1}={\tilde{\sigma }}_{xx}-{\tilde{\sigma }}_{yy}$ at $\tilde{x}=-75$. The selected cross-section is so that the effects of the inlet and the contraction region can be neglected. In these plots, $\tilde{y}=0$ and 4 represent the centerline and the wall, respectively. In Figure 5a, the profiles of the axial velocity are shown. Note that the value of De is evaluated at the outlet; therefore, the Deborah number calculated at the upstream using the inlet velocity and the inlet width is only 1/16 of the written value. This explains why there is no evidence of shear banding for these small values of De before the contraction. The profiles of the polymer number density are shown in Figure 5b. The overshoot generated at larger De is due to the Fickian diffusion, which moves toward the wall as De is increased. In Figure 5c, the profiles of the shear stress are depicted. The value of ${\tilde{\sigma }}_{xy}$ is zero at the centerline and increases linearly to the maximum value at the wall, as typically observed for pressure-driven channel flow [25]. The absolute wall shear stress is larger for larger De numbers. In Figure 5d, we show the first normal stress difference, which is larger for a flow with increasing values of De. The value of ${\tilde{N}}_{1}$ is zero at the centerline and quadratically increases for larger De as the wall is approached.

**Figure 6:**
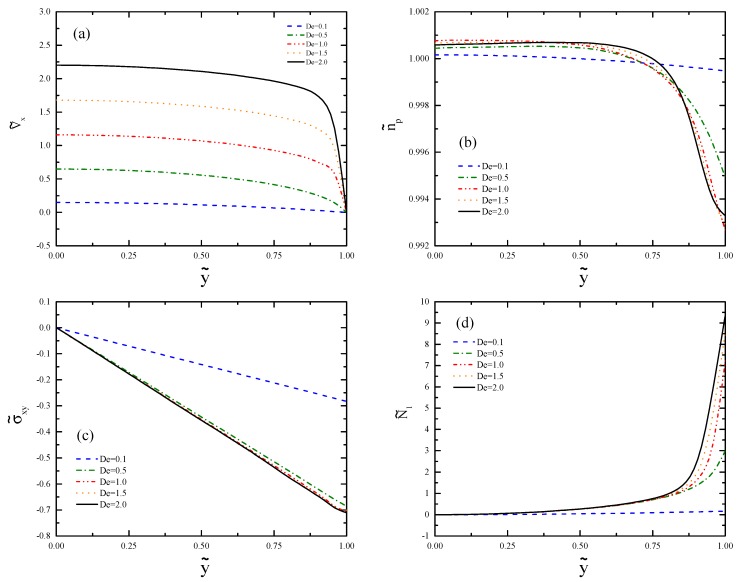
Profiles of (**a**) axial velocity, (**b**) polymer number density, (**c**) shear stress, and (**d**) first normal stress difference evaluated at $\tilde{x}=75$ for different Deborah numbers.

Figure 6 shows the results of the channel far away from the contraction at $\tilde{x}=75$. The velocity profiles shown in Figure 6a deviate from the typical parabolic profile of channel flow and form plug-like flow for De⩾1. This phenomenon is related to shear banding. The kink separating the bands move toward the center of the channel as De increases. The profiles of the polymer number density are illustrated in Figure 6b. For De⩾1, we can see clear bands where the band near the centerline forms a plateau, and the other band shows a strong decrease near the wall. The effect of shear banding is much smaller than the effect of recirculation for the De values considered in this work (Figure 6b). However, at very large De values, the opposite trend may be found. Figure 6c shows that the profiles of the shear stress increase from zero at the centerline to the extremum at the wall. The overlap of these profiles in the shear banding regime is expected from the plug-like velocity profiles, where the similar shear rates of the bands result in similar stress profiles in the plateau regime of the flow curve. The small difference in the bands arises from the relatively narrow range of De examined. The profile of the shear stress in the shear banding regime shows nonlinearity near the wall if the resolution of the mesh is not high enough; therefore, we used the mesh M4 to obtain Figure 6c.

**Figure 7:**
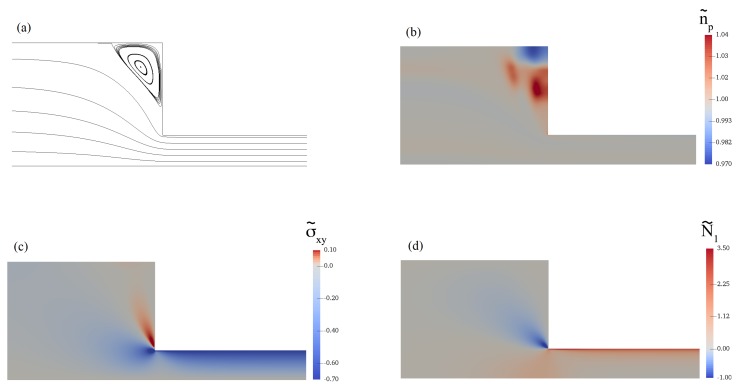
Contours of (**a**) stream function, (**b**) polymer number density, (**c**) shear stress, and (**d**) first normal stress difference for De=0.5.

**Figure 8:**
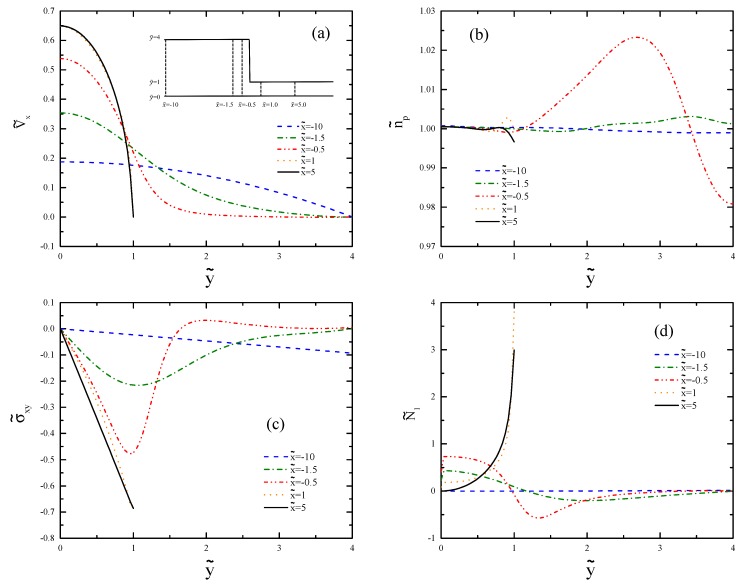
Profiles of (**a**) axial velocity, (**b**) polymer number density, (**c**) shear stress, and (**d**) first normal stress difference for De=0.5 at vertical cross-sections $\tilde{x}=-10$, −1.5, −0.5, 1.0, and 5.0.

**Figure 9:**
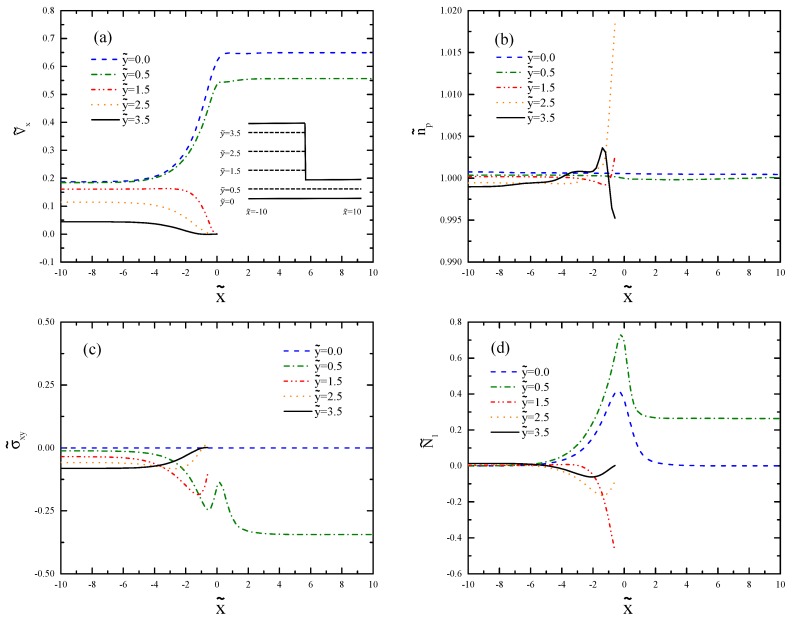
Profiles of (**a**) axial velocity, (**b**) polymer number density, (**c**) shear stress, and (**d**) first normal stress difference for De=0.5 at horizontal cross-sections $\tilde{y}=0$, 0.5, 1.5, 2.5, and 3.5.

Figure 7 shows the streamlines and the contours of the flow for De=0.5. We can see a vortex at the corner in Figure 7a. The contour of the polymer number density is shown in Figure 7b. We note a huge increase in ${\tilde{n}}_{p}$ as a result of flow recirculation. At the re-entrant corner, there is a concentrated zone of large absolute shear stress values (Figure 7c). The first normal stress difference (Figure 7d) becomes negative as a result of a large ${\tilde{\sigma }}_{yy}$ value. It must be noted that ${\tilde{\sigma }}_{zz}$ is nonzero in the channel, although the values are much smaller than the other normal components of the shear stress. Our viscoelastic flow predictions depicted in Figure 7, Figure 8 and Figure 9 qualitatively agree with the velocity and stress calculations of the Giesekus, PTT, and FENE-P models [33,34].

Figure 8 shows the results for the flow De=0.5 at different vertical cross-sections before and after the contraction. We see in Figure 8a how the axial velocity profile adapts itself to the narrow channel. The velocity strongly increases due to the requirement of the mass conservation. However, the shear rate is still not large enough for De=0.5 to form a banded plug-like curve. Figure 8b shows the profiles of the polymer number density in the vertical sections. The strong overshoot of the profile at $\tilde{x}=-0.5$ corresponds to the recirculation region depicted in Figure 7b. In Figure 8c, we can see that the value of the shear stress changes its sign in the recirculation region, while this profile is linear further away ($\tilde{x}=-10$ and 5). The profiles of the first normal stress difference are shown in Figure 8d. The negative sign of ${\tilde{N}}_{1}$ in the recirculation zone suggests that the ${\tilde{\sigma }}_{yy}$-component dominates in this region. The large values of the first normal stress difference after the contraction region is due to the larger local De after the contraction.

Figure 9 shows the profiles in cross-sections parallel to the centerline. Since the important changes happen in the contraction region, we only depict the part of the channel in the range $-10⩽\tilde{x}⩽10$. There is strong shearing near the walls and significant uniaxial extension along the centerline. As the flow approaches the contraction, the increasing extension rate increases the velocity for $\tilde{y}=0$ and 0.5 (Figure 9a). It is evident from Figure 9b that ${\tilde{n}}_{p}$ increases in the recirculation region at $\tilde{y}=2.5$ and overshoots at $\tilde{y}=3.5$, which matches with the shape of the vortex. We can see in Figure 9c that ${\tilde{\sigma }}_{xy}$ undershoots and overshoots before and after the contraction, respectively. The maximum of ${\tilde{N}}_{1}$ occurs close to the contraction (Figure 9d).

**Figure 10:**
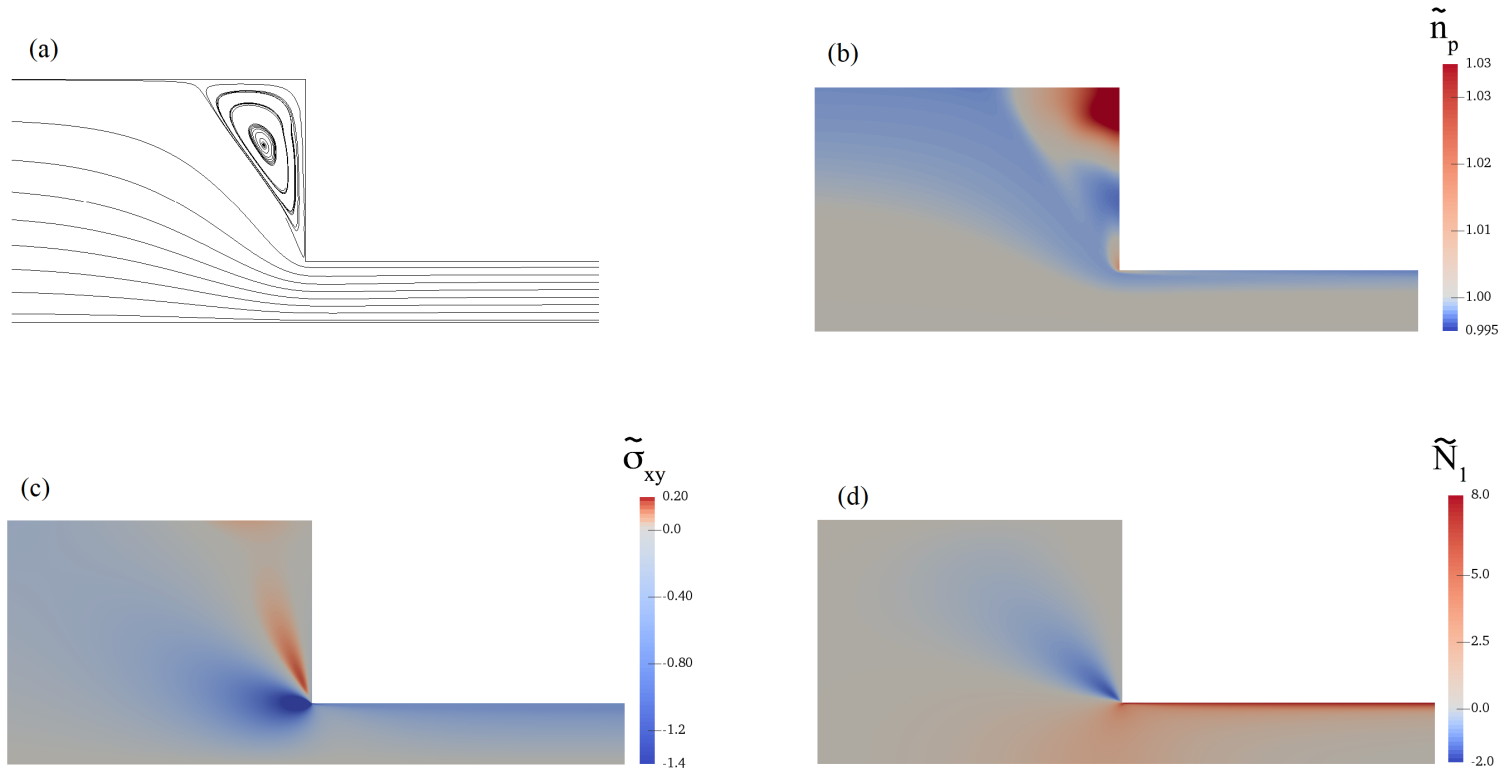
Contours of (**a**) stream function, (**b**) polymer number density, (**c**) shear stress, and (**d**) first normal stress difference for De=2.0.

The streamlines and contours are shown for De=2 in Figure 10 and are compared with the information of Figure 7. The vortex size is larger, and the accumulation of the polymer particles in the recirculation zone is closer to the wall. The contours of the shear stress and first normal stress difference are qualitatively similar, but the magnitudes are larger.

**Figure 11:**
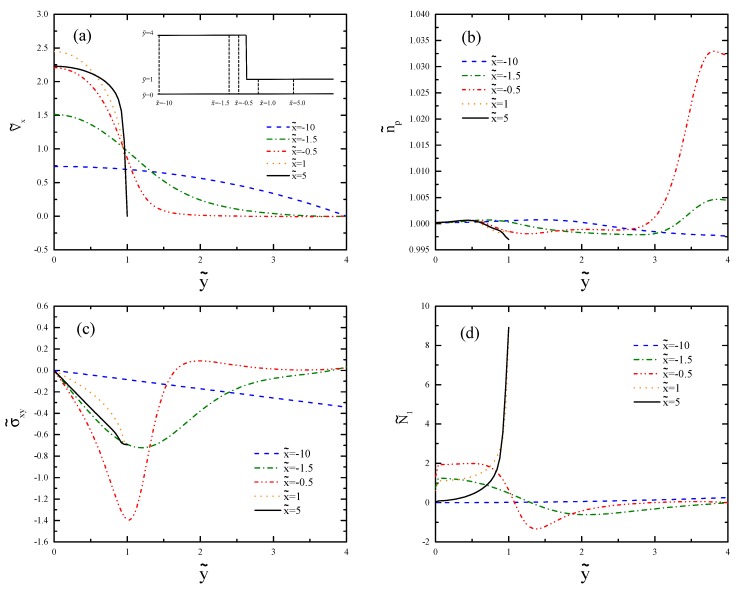
Cross-sectional profiles of (**a**) axial velocity, (**b**) polymer number density, (**c**) shear stress, and (**d**) first normal stress difference for De=2.0. The selected $\tilde{x}$-values are the same as in Figure 8.

Figure 11 shows the profiles for De=2 at different vertical cross-sections of the channel before and after the contraction region. The results are compared with those obtained from Figure 8. In Figure 11a, we can see the transition from parabolic flow to plug-like flow as the fluid flows from small deformation regime at the wide channel to the shear banding regime at the narrow channel. The increased polymer number density in the contraction region (Figure 11b) is closer to the wall because of the larger De value. The shear stress profiles (Figure 11c) qualitatively follow the same trend; however, the values are larger. The sharp profile of ${\tilde{N}}_{1}$ (Figure 11d) is due to the shear band formation.

**Figure 12:**
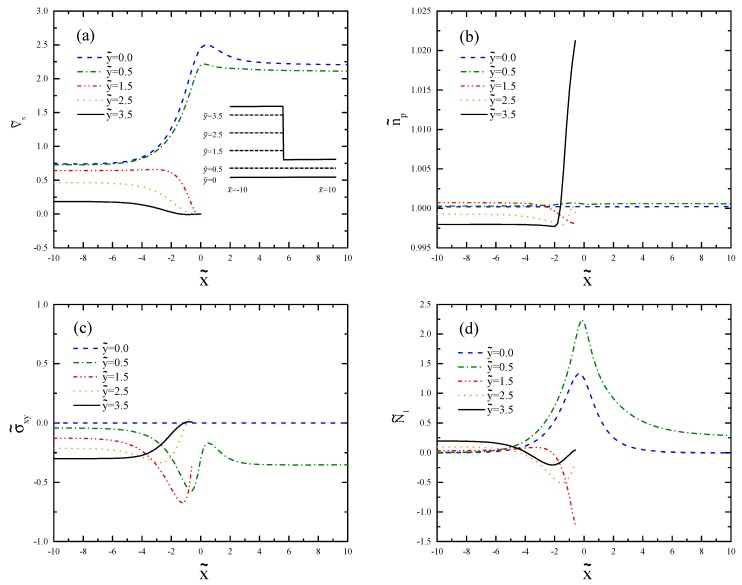
Cross-sectional profiles of (**a**) axial velocity, (**b**) polymer number density, (**c**) shear stress, and (**d**) first normal stress difference for De=2.0. The selected $\tilde{y}$-values are the same as in Figure 9.

Figure 12 shows how the profiles change near the contraction region in different horizontal cross-sections. There is an overshoot of the axial velocity, as shown by Kim et al. [35] for larger De. We see in Figure 12c that ${\tilde{\sigma }}_{xy}$ undershoots before the contraction and overshoots after it, as already seen in Figure 9 for De=0.5.

## 5. Conclusions

We studied shear banding of semidilute entangled polymer solutions in 4:1 planar contraction flow using our recently developed two-fluid model. The model was derived using the generalized bracket approach of nonequilibrium thermodynamics. It is based on the hypotheses that diffusional processes trigger the observed steady-state shear banding. As opposed to the standard one-fluid polymer models, our model can predict not only steady-state velocity banding but also banded concentration profiles. We expect that standard one-fluid models with/without polymer migration could also predict the velocity bands in the 4:1 geometry. However, because of the missing velocity-concentration-stress coupling, concentration banding could not be predicted. This quantity is of major relevance to industrial processing as it directly affects the texture of the material.

The results of the contraction flow reveal that the size and the intensity of the corner vortex increase with the Deborah number as a result of shear-thinning, but they decrease after the onset of shear banding; these findings are in agreement with experiments. The axial velocity profile forms a plug-like shape in the shear banding regime after the contraction, where the local De number is much larger because of the increased velocity. The kinks separating the velocity bands move toward the centerline as De increases. For the De values investigated in this work, the concentration of the polymer in the recirculation region is strongly increased. However, there is an increased effect of shear banding on ${\tilde{n}}_{p}$ at larger De, typically encountered in industrial processing flows.

The simplicity of the new model and the results encourage us to study more complex phenomena, such as die swell of the polymer solutions in extrusion flow. Furthermore, additional experiments are needed to quantitatively compare their results with the predictions of the new model.

## Figures and Tables

**Table 1:** Mesh characteristics.

Mesh	$\Delta x_{min}/H=\Delta y_{min}/H$	Number of Cells
M1	0.0042	6051
M2	0.0021	24,204
M3	0.0014	54,459
M4	0.0007	217,836

## Appendix A

The dimensionless forms of Equations (1)–(7) using the scalings introduced in Section 2 are as follows:

(A1) $$\begin{array}{c} E^{-1}\frac{\partial \tilde{v}}{\partial \tilde{t}}=-E^{-1}\tilde{v}\;\cdot \;\tilde{\nabla }\tilde{v}-\tilde{\nabla }\tilde{p}+\tilde{\nabla }\;\cdot \;\tilde{\sigma }\;, \end{array}$$

(A2) $$\begin{array}{c} \tilde{\Delta v}=\frac{1}{\tilde{D}}\left[\frac{\chi {\tilde{n}}_{s}}{{\tilde{n}}_{p}+\chi {\tilde{n}}_{s}}\left\{-\tilde{\nabla }\left({\tilde{n}}_{p}\right)+\tilde{\nabla }\;\cdot \;\tilde{\sigma }\right\}+\frac{{\tilde{n}}_{p}}{{\tilde{n}}_{p}+\chi {\tilde{n}}_{s}}\left\{\tilde{\nabla }\left({\tilde{n}}_{s}\right)+\beta {\tilde{\nabla }}^{2}{\tilde{v}}^{s}\right\}\right]\;, \end{array}$$

(A3) $$\begin{array}{c} \frac{\partial {\tilde{n}}_{p}}{\partial \tilde{t}}=-\tilde{\nabla }\;\cdot \;\left({\tilde{v}}^{p}{\tilde{n}}_{p}\right)\;, \end{array}$$

(A4) $$\begin{array}{cc} \frac{\partial \tilde{C}}{\partial \tilde{t}}= & -\tilde{\nabla }\;\cdot \;\left({\tilde{v}}^{p}\tilde{C}\right)+\tilde{C}\;\cdot \;\tilde{\nabla }{\tilde{v}}^{p}+{\left(\tilde{\nabla }{\tilde{v}}^{p}\right)}^{T}\;\cdot \;\tilde{C}\\ & -\left[\left(1-\alpha \right)I+\alpha \tilde{C}\right]\left(\tilde{C}-I\right)\\ & +\epsilon {\left(\mathrm{tr}\tilde{C}-3\right)}^{q}\left(\tilde{C}-I\right)\\ & +{\tilde{D}}_{nonloc}\left(\tilde{C}\;\cdot \;\tilde{\nabla }\left(\tilde{\nabla }\;\cdot \;{\tilde{\sigma }}^{p}\right)+{\left[\tilde{\nabla }\left(\tilde{\nabla }\;\cdot \;{\tilde{\sigma }}^{p}\right)\right]}^{T}\;\cdot \;\tilde{C}\right) \end{array}$$

(A5) $$\begin{array}{c} \tilde{\sigma }=\tilde{C}-{\tilde{n}}_{p}I+\beta \left[\tilde{\nabla }{\tilde{v}}^{s}+{\left(\tilde{\nabla }{\tilde{v}}^{s}\right)}^{T}\right], \end{array}$$

(A6) $$\begin{array}{c} {\tilde{v}}^{p}=\tilde{v}+\frac{\chi {\tilde{n}}_{s}}{{\tilde{n}}_{p}+\chi {\tilde{n}}_{s}}\tilde{\Delta v}\;, \end{array}$$

(A7) $$\begin{array}{c} {\tilde{v}}^{s}=\tilde{v}-\frac{{\tilde{n}}_{p}}{{\tilde{n}}_{p}+\chi {\tilde{n}}_{s}}\tilde{\Delta v}. \end{array}$$
